# 

**Published:** 1893-03-25

**Authors:** 


					410 THE HOSPITAL Mabch 25, 1893.
OUR NEW OFFICES.
428, Strand, W.C., will henceforth bo the Office of this Journal,
NOTICE TO CORRESPONDENTS.
All MS., Letters, Books for Review, and other matters intended foithe
Editor should he addressed Thx Editob, Tee Lodge, Pouohebteb
Square.Condon, W.
The Editor cannot undertake to return rejected U.S., even when
aosompanied by stamped direoted envelope.
Notice to Advertisers and Subscribers.
All Advertisements, Orders for Oopies of The Hospital, and Business
Communications should be addressed to The Publisher, not to the Editor,
The Hospital, 428, 8trand, W.O.
The Cost of Subscription, post free, is as followsPer week, 2id. {
per quarter, 2s. 6d. ; per half-year, 5s. ; per annum, 8s. 6d,
ForeignPer annum, 10s. 8d.; payable, in all c&ses, in advance.
" Burdett's Hospital Annual," 1893.
Fourth year of publication. 700 pp. Boards, gilt lettering. A most
complete guide to British, Colonial, Indian, and American Hospitals,
Dispensaries, Nursing ?nd Convalescent Institutions, and Asylums.
Price5s. Published by The Scientific Press (Limited), 423, Strand,
W.O.
NOTICE.?The Index for Vol. XII. fending1 September
1892) is on sale at tlie Office of this Journal, price
2$d., post free.
Scraps and Gleanings.
A balance of ?226 8s. is reported by the treasurer of the Biceford
Dispensary.
It is tr-3eot:d lhat the new portion of the Bnry Hospital will be ready
tor patients in May.
Italian express trains have now been beaten in their time record by
the fast Atlantic steamboats.
The fai nre of Head's Baiik at the commencement of lait year proved
a loss of ?175 to the East Uriustead Cottage Hospital,
The United Kingdom last year imported oranges and lemons, which
were valued at the apparently high figure of ?2,052,561.
The local ball in aid of the Wrexham Infiimary proved a great tuo-
cess, the financial results of which realised about ?120.
The Lower House of Hungary has passed a Bill granting a fixed-
salary of 2,400 fljrins a year to its Parliamentary membsrs.
The authorities of Staleybridge, Dunkerfield, and Hyde are meeting;
with the view of erecting a joint infections diseases hospital.
For the Book World of Medicine and Science, and Student of Medicine and Medical Appointments, see pages
Vill., IX? X., and 2EI. overleaf.
Books Received.
Ghatto and Windus.
" The Shilling House of Commons for 1893."
" The Shilling Peerage for 1893."
Oliver and Boyd,
" Cheyne-Stokes Respiration," By George A, Gibaon, M.D.
Bailliere, Tindall, and Ook.
" Notes on Medicinal Remedies." By J. B> Stephenson,
Simpkin, Marshall, and Go,
"The Medical Annual, 1893,"
x THE HUSP11AL. March 25, 1893.
The Student of Medicine.
iAll communications lor this Supplement should be addressed to Thh Editob of Thb Hospital, at 428, Btrand, with tJia words," Student*
Column," written in the left hand top corner of the envelope.!
APPOINTMENTS.
James Alexander, M.B., C.M., appointed Medical Officer for
the Galston Parochial Board. George Barnes, L.R.C.P.Edin.,
M.R.C.S.Eng., appointed Medical Officer of Health for Chard
Urban Sanitary District. Charles Wooiiscroft Belfield, M.D.
Vienna, L.R.C.P.Edin., M.R.C.S.Eng., appointed Medical Officer
to the No. 2 District of the Bristol Union. A. A. Blakiston,
M.R.C.S.Eng., L.S.A., reappointed Medical Officer of Health
for the Borough of Glastonbury. H. Broadhurst, M.R.C.S.,
L.R.C.P.Lond., appointed Resident Clinical Assistant to the St.
Marylebone Infirmary, Not ting Hill. Thos. Callaghan, L.R.C.P.,
L.R.C.S.Edin., L.F.P.S.Glasg., appointed an Extern-Physician
to the Cork Fever Hospital. Mr. A. D. Carse, appointed Medical
Officer of the Scotland District of the Rochdale Union. P. A.
Colmer. M.R.C.S., L.R.C.P.Lond., L.S.A., appointed Honorary
Medical Officer to the Yeovil District Hospital. James Donelan,
M.B., M.Ch.Univ.Irel., appointed Honorary Physician to the
Italian Hospital, Queen Square, W.C. Robert Bruce Duncan,
M.B., B.S.Durh., appointed Visiting Medical Assistant to the
Newcastle-on-Tyne Dispensary. Chas. Gordon Gibson, M.B.,
C.M.Edin., reappointed Medical Officer of Health for the
Launceston Rural Sanitary District. Arthur E. Giles, M.D.,
B.Sc.Lond., M.R.C.P., &c., appointed Physician-Accoucheur to
the St. Marylebone Dispensary, Welbeck Street, W. John Kenaz
Goodall, L.R.C.P., L.R.C.S.Edin., reappointed Medical Officer
of Health for Whittington Urban Sanitary District. John
Griffith, M.R.C.S.Eng., L.R.C.P.Lond., appointed Pathologist
and Curator to the Royal Westminster Ophthalmic Hospital,
King "William Street, Strand, W.C. Matthew Corri Halton,
L.R.C.P.I., L.M., appointed Surgeon to the Barnsley Post Office.
Nathan Hannah, L.R.C.P.Edin., L.F.P.S.Glasg., reappointed
Medical Officer of Health for the Abram Urban Sanitary District.
Arthur Hooley, L.R.C.P.,Edin? M.R.C.S., appointed District
Medical Officer of the Epsom Union. A. B. How, L.R.C.P.
Lond., M.R.C.S.Eng., appointed Medical Officer of the Strad-
brokeDistrict of the Hoxne Union. Wm. Rees Jones, M.D.Glasg.,
M.B., C.M., reappointed District Medical Officer of the Brecon
Union. P. C. Karkeek, M.E.C.S., reappointed Mecucal ymcw ?*
Health for St. Mary church, Torquay. Chas. Marshall Kemp >
M.E.C.S.Eng., reappointed Medical Officer of Health for on?
ham. Alfred Wm. Laing, L.E.C.P.Lond., M.E.C.S., appoiQ _
Medical Officer of the No. 7 District of the Tonbridge Uniou.
Mr. J. H. Littlejohn, appointed Medical Officer for the Mans
District of the Ludlow Union. James H. Lloyd, M.E.C.b.iwe,?
L.E.C.P.Edin., appointed Certifying Surgeon for the Collamp
District, Devon, under the Factory and Workshops Act. w. ?
M. Lowe, M.B., Ch.B.Vict.Univ., appointed Junior House a
geon to the Wigan Infirmary. W. Stewart McDougall, M. ??
C.M.Edin., appointed Medical Officer of the Parish of Tong '
Sutherlandshire. J. Menzies, L.E.C.P., L.M., L.E.C.S.Ed ??
L.F.P. and S.Glasg., appointed Medical Officer of Health to
Shireoaks Sick Club, Worksop. J. Murphy, M.A.(>M.D-i ^
pointed Lecturer in Medical Jurisprudence in the University
Durham College of Medicine at Newcastle-on-Tyne. T. My1 ?
M.B., appointed Medical Officer of the Iveley District ox
Wigton Union. Newby, Gervase Edward, M.E.C.S., L.K.y- '
Lond., appointed House Surgeon to the Salford Eoyal Hospi ?
Frederick J. Parsons, M.E.C.S.Eng., reappointed Medical Otu
of Health for the Frome Urban Sanitary District. J. y. j
Watkin Penney, M.B., C.M.Glasg., appointed District Meo*
Officer for_ County Bute. Sidney Herbert Perry, M.B
M.E.C.S., L.E.C.P., appointed House-Physician to the Gene
Hospital, Birmingham. John Wyatt Pratt, L.E.C.P., L.M.
M.E.C.S., reappointed Medical Officer of Health J0T? ag
Wiveliscombe Urban Sanitary District. David Valent?
L.E.C.P.Lond., M.E.C.S.Eng., reappointed District
Officer of the Brecon Union. E. N. Eeichhardt, 5'
appointed District Medical Officer of the Epsom Union
i-U-i XV ? v ? kj? j icuypuiub&u iu.ouiuuii w* f T? oflS
Wiveliscombe Urban Sanitary District. David Valentine we j
Ti.R.n.P.Tjond.. M.E.O.S.Enff.. reannnintad District Meai,
Lond.t
lerna^d
Eice, M.D.Lond., M.E.C.S., appointed Police Surgeon *?rJjj0
Borough of Leamington. W. Eichardson Eice, B.A.,
M.D.Univ.Dublin, appointed Examiner to the St. JohnAffl^j.
lance Association. Mr. William Selby, appointed Medical 0?.^
of the Workhouse of the Parish of Birmingham. W.
Shaw, M.E.C.S.Eng., appointed Medical Officer of the Wo
house of the Fylde District. T. F. Hugh Smith, F.R-^' *'
^arch 25, 1893. THE HOSPITAL.
XI
THE STUDENT OF MEDICINE?(continued).
?     ?
Un ' ?> aPPointed Medical Officer to the Hospital Convalescent
ao*e, Parkwood, Swanley.
T VACANCIES.
lalaJl .'?^?wing vacancies are announoed thia week, the amount of
in each case being given after the name of the institution t
0, Resident Medical Offloer*.
,kire County Asylum, Macclesfield. Junior Assistant Medical
Officer; donbly qualified and unmarried. Salary ?105 per annum,
^isinjf to ?125 by two yearly advances of ?10, with board, washing,
yii^Tc. lodging. Applications to the Medical Superintendent.
"""hire Dispensary, Bagills Street, Holywell. Resident House
Surgeon. Salary ?120 per annum, with furnished house, rent, and
*o^eB *ree, alio coal, light, water, and cleaning, or in lieu thereof
, 0 tier annum. Applications to William Thos. Oole, Secretary, by
G April 11th.
"eneral Hospital, Birmingham.?Assistant House Surgeon. Board,
?esidenco, and washing provided. Applications to Howard J.
B* Ho*se (Jovernor, by April 1st.
?organshire and Monmouthshire Infirmary, Cardiff. Assistant
blouse Snrgeon. Appointment for six months. Board, lodging, and
provided. Applications to George T. Oolman, Secretary
Leithri ??^eral Superintendent, by March 81st.
/. hospital. House Physician, Honse Burgeon, and Snrgeon for the
outdoor Department. Appointments for six months, and tne
salary attached to eaoh office is at the rate of ?50 per annum, with
ooard. Applications to Mr. George V. Mann, Secretary, 33, Bernard
iWo^^keith, by March 31st. , ,.
r.Jw?et General Dispensary and Accident Ward, Pontefract. Medi-
Officer; donbly qualified. Salary ?130 per annum, with furnished
eonis, fire, lights, and attendance. Applications to Percy r.
HovaiTT* Secretary, by April 7th. ,, ,
rtl See Hospital, Gray's Inn Road, W.O. Senior Medical Offioer;
y Qualified. Salary ?100 per annum, with board, residence,
Hoyaf^Jvaa5tin?*. Applications by April 17th.
?w. ^ree Hospital, Gray's Inn Road, W.O. Junior Medical Officer.
^8t^e duly registered. Board, residence, and washing. Appuoa-
Children's Homital (East End Branch), Sheffield. House
B^geon; doubly qualified. Salary ?70 per annum, with board,
v eing*an<l washing. Applications to the Honorary Secretary by
?cl1 30th.
London Hospital, Hammersmith Road, W. House (Surgeon;
appointment for six months. Board and lodging provided. Appli-
cation, to R. J. Gilbert, Secretary Superintendent, by March 22nd.
Election on March 27th.
Victoria Hospital for Sick Children, Hull. Resident Medical Officer.
Salary ?50 per annum, with board, rooms, and washing. Applica.
tions to J. Travis Cook, Honorary Secretary, 14, Parliament Street,
Hull, by April 3rd.
Worcester General Infirmary. House Surgeon; doubly qualified.
Salary ?100 per annum, with board and residence. Appointment
for three years. Applications to William Stallard, Secretary,
Worcester Chambers, Pierpoint Street, Worcester, by March 25th.
tfon-Besldent Medical Officers.
Bolton Infirmary and Dispensary. Senior House Surgeon; doubly
qualified; age not to exceed 30. Salary ?120 per annum, with
furnished apartments, board, and attendance. ApDlioations to Peter
Kevan, Esq., Honorary Secretary, 12, Acresfield, Bolton, by March
28th.
Brighton Throat and Ear Hospital, 23, Queen's Boai, Brighton. Non-
resident Houte Surgeon. Salary at the rate of ?50 per annum.
Applications to the Secretary by March 29th.
General Hospital, Birmingham. Assistant Surgaon. Honorarium, ?100
per annum. Applications to Howard J. Collins, House Governor,
by April 1st.
General Hospital, Birmingham. Pathologist. Salary ?120Jper annum.
Applications to Howard J. Collins. House Governor, by April 1st.
London Hospital, Whitechapel Road, E. Assistant Phygioian, Appli-
cations to the Hou?e Governor by March 25th.
Mason College. Birmingham. Co-Professor of Surgery. Applications
to G. H. Morley, by Aoril 15th.
Metropolitan Hospital, Kingsland Road, N.E. House Phyrician;
doubly qualified. Appointment for six months. Salary at the rate
of ?60 per annum. Applications to Charles H, Byers, Secretary, by
March 25th.
Metropolitan Hospital, Kingsland Road, N.E. House Surgeon ; doubly
qualified. Appointment for six months. Salary at the rate of ?60
per annum. Applications to Charles H. Byers, Seoretary, by
March 25th.
Metropolitan Hospital, Kingsland Road, N.E. Assistant House Sur-
geon ; doubly qualified. Appointment for six months. Applications
to Charles H. Byers, Secretary, by March 25tb.
New Hospital for Women, 144, Euston Road, N.W. Qualified medical
woman as Clinical Assistant. Appointment for six months. Appli-
cations to Margaret M. Bogster. Seoretary, by March 27th.
Royal College of Physicians, Pall Mall East, S.W. Milroy Lecturer.
Applications to the Registrar by April 5th,
South Devon and East Cornwall Hospital, Plymonth. Assistant
Secretary; not under 35 years of age. Salary ?150 per annum.
Applications to J.Walter,Wilson, Honorary Secretary, by Maroh 25th.
Sunderland Infirmary. Honorary Surgeon. Applications to the Chair-
man of the Medical Board by April 6th.

				

